# 4S-fluorination of ProB29 in insulin lispro slows fibril formation

**DOI:** 10.1016/j.jbc.2024.107332

**Published:** 2024-05-03

**Authors:** Stephanie L. Breunig, Alex M. Chapman, Jeanne LeBon, Janine C. Quijano, Maduni Ranasinghe, Jeffrey Rawson, Borries Demeler, Hsun Teresa Ku, David A. Tirrell

**Affiliations:** 1 Division of Chemistry and Chemical Engineering, California Institute of Technology, Pasadena, California, USA; 2 Department of Translational Research and Cellular Therapeutics, Arthur Riggs Diabetes and Metabolism Research Institute, Beckman Research Institute City of Hope, Duarte, California, USA; 3 Department of Chemistry and Biochemistry, University of Lethbridge, Lethbridge, Alberta, Canada; 4 Department of Chemistry and Biochemistry, University of Montana, Missoula, Montana, USA; 5 Irell & Manella Graduate School of Biological Science, City of Hope, Duarte, California, USA

**Keywords:** non-canonical amino acid, fluoroproline, proline, insulin, insulin lispro, fibrillation

## Abstract

Recombinant insulin is a life-saving therapeutic for millions of patients affected by diabetes mellitus. Standard mutagenesis has led to insulin variants with improved control of blood glucose; for instance, the fast-acting insulin lispro contains two point mutations that suppress dimer formation and expedite absorption. However, insulins undergo irreversible denaturation, a process accelerated for the insulin monomer. Here we replace ProB29 of insulin lispro with 4*R*-fluoroproline, 4*S*-fluoroproline, and 4,4-difluoroproline. All three fluorinated lispro variants reduce blood glucose in diabetic mice, exhibit similar secondary structure as measured by CD, and rapidly dissociate from the zinc- and resorcinol-bound hexamer upon dilution. Notably, however, we find that 4*S*-fluorination of ProB29 delays the formation of undesired insulin fibrils that can accumulate at the injection site *in vivo* and can complicate insulin production and storage. These results demonstrate how subtle molecular changes achieved through non-canonical amino acid mutagenesis can improve the stability of protein therapeutics.

Insulin, a 5.8 kDa peptide hormone composed of two disulfide-linked chains (A and B), is a widely-used, essential therapeutic for individuals with diabetes mellitus. Insulin is normally secreted from pancreatic β-cells in response to elevated blood glucose (1). Diabetes, which affects more than 400 million adults world-wide (https://www.who.int/publications/i/item/9789241565257), arises from dysfunction in insulin signaling, either from loss of insulin secretion (type 1) or from development of insulin resistance (type 2). Advances in insulin technology over the last century, notably the recombinant production of insulin (2), have dramatically reduced the mortality rate associated with diabetes. Individuals with type 1 diabetes typically rely on insulin replacement therapy to compensate for loss of pancreatic function, while those with type 2 often supplement lifestyle changes with oral medications that enhance insulin signaling and, in severe cases, receive exogenous insulin.

In its pharmaceutical formulations, insulin exists as a zinc- and phenolic ligand-bound hexamer (referred to as the R_6_ state) (3). Upon subcutaneous injection, insulin hexamers dissociate through lower-order oligomeric species before crossing the capillary membrane in the active monomeric state (4, 5) (Fig. 1*A*). Because dissociation to the monomer is rate-limiting for insulin absorption into the bloodstream, disrupting association also accelerates onset of action (6). Introducing point mutations that disfavor oligomer formation has resulted in FDA-approved fast-acting insulins (FAIs) (7, 8, 9). FAIs are rapidly released into the bloodstream (10), approaching the transient increases in insulin concentration stimulated by elevated blood glucose. Typical insulin replacement therapies combine regular treatments of basal (long-acting) insulin variants with injections of FAIs before mealtime to approximate the insulin-action profile of a healthy pancreas (10). However, insulins are prone to chemical and physical denaturation, such as the irreversible formation of amyloid fibrils. Insulin fibrils can accumulate *in vivo* at sites of repeated injection and complicate industrial production, distribution, and storage (11). FAIs are more prone than human insulin to chemical and physical denaturation, since the protective effects of oligomerization are interrupted (12).

**Figure 1:**
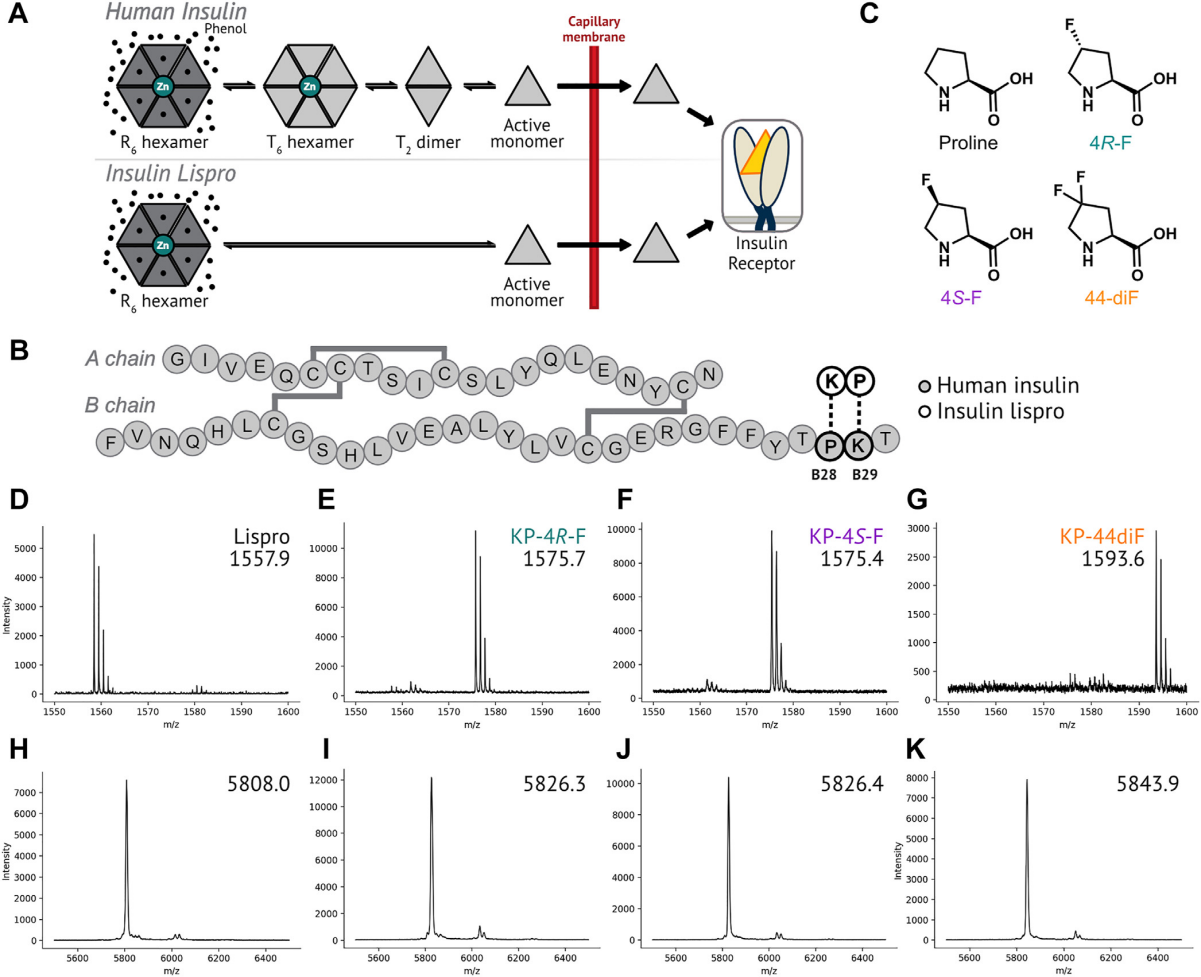
**Proline mutagenesis at position B29 of insulin lispro.***A*, simplified representation of insulin and insulin lispro oligomerization. Disrupted oligomerization speeds the release of lispro into the bloodstream. *B*, the amino acid sequences of human insulin and insulin lispro. *C*, the structures of proline and the proline analogs used in this study. *D*–*G*, characterization of proline analog incorporation. Proinsulin was expressed in media supplemented with proline (*D*), 4*R*-F (*E*), 4*S*-F (*F*), 44-diF (*G*), digested with Glu-C, and analyzed by MALDI-TOF MS. The peptide-containing position B29 of mature lispro is ^50^RGFFYTK**P**TRRE (expected m/z = 1557.8). *H*–*K*, MALDI-TOF characterization of mature insulin variants: insulin lispro (*H*), KP-4*R*-F (*I*), KP-4*S*-F (*J*), and KP-44-diF (*K*). The peaks at m/z ∼6050 correspond to adducts of the sinapic acid matrix. 44-diF, 4,4-difluoroproline; 4R-F, 4R-fluoroproline; 4S-F, 4S-fluoroproline.

Insulin lispro (Humalog, Eli Lilly), the first commercially available FAI, is characterized by reduced monomer association as a consequence of the inversion of residues ProB28 and LysB29 (Fig. 1*B*) (7). Removal of the constrained pyrrolidine ring of proline at position B28 grants additional conformational degrees of freedom and removes hydrophobic packing interactions at the dimer interface. These modifications disrupt the homodimer interaction (7) and accelerate absorption (6). However, the standard mutagenesis approach employed in the discovery of insulin lispro (7) (and other FDA-approved insulin variants (8, 9, 13)) is limited by the chemical functionality available in the 20 canonical amino acids and restricted with respect to the chemical space that can be explored. In particular, no other canonical amino acid can recapitulate the unique structural and conformational properties of proline (14). In contrast, protein engineering strategies that draw upon non-canonical amino acids afford access to chemistries beyond those found in natural or conventionally-engineered proteins (15).

Non-canonical proline (ncPro) residues provide chemical diversity while maintaining proline’s cyclic structure. Many ncPro residues exhibit conformational biases different from those of proline (14) and have been used to interrogate the importance of proline conformation in protein behavior. Proline analogs with 4*R* electron-withdrawing groups (such as 4*R*-fluoroproline, 4*R*-F; Fig. 1*C*) promote a C^γ^-*exo* ring pucker due to a gauche effect, which in turn enforces the *trans* amide isomer. Conversely, ncPro residues with 4*S*-electron–withdrawing groups (such as 4*S*-fluoroproline, 4*S*-F) favor the C^γ^-*endo* pucker and the *cis* amide isomer, compared to canonical proline (14). The position of the amide *cis*-*trans* equilibrium of 4,4-difluoroproline (44-diF) is similar to that of proline (16). Isomerization of the fluorinated analogs is accelerated relative to proline, as an inductive effect reduces the bond order of the preceding amide (16). Proline analogs have been used to determine the stereoelectronic origin of collagen stability (17), to identify a key *cis*-*trans* isomerization event in the opening of the 5-HT_3_ receptor (18), to probe the role of *cis-trans* isomerization in β2 microglobulin fibrillation (19), and to modify protein stability (16, 20, 21, 22).

We recently demonstrated that introduction of ncPro residues at position B28 can be used to tune the biophysical properties of human insulin (23, 24, 25). These results prompted us to ask whether similar changes in the biophysical properties of insulin lispro might be achieved. To explore this question, we replaced ProB29 in insulin lispro with 4-fluorinated proline analogs (Fig. 1*C*); these lispro variants will be referred to as KP-4*R*-F, KP-4*S*-F, and KP-44-diF (KP refers to the inversion of the positions of the lysine (K) and proline (P) residues in the lispro sequence).

## Results

Lispro variants were expressed in *Escherichia coli* as the corresponding proinsulins (precursors to insulin) under conditions that favor ncPro incorporation (26) at position B29. Briefly, the proline auxotrophic strain CAG18515, engineered to overexpress the *E. coli* prolyl-tRNA synthetase, was grown in M9 medium supplemented with all 20 amino acids until late log phase. Cells were washed and resuspended in a medium lacking proline, at which point 0.3 to 0.5 M NaCl (to facilitate proline uptake) and the proline analog (0.5 mM) were added. The extent of proline replacement was assessed by MALDI-TOF mass spectrometry of a proinsulin peptide fragment (Fig. 1, *D*–*G*) and of mature insulin (Fig. 1, *H*–*K*). Incorporation of all of the fluorinated proline analogs exceeded 90% (Table S1). The yield of proinsulin-lispro in rich medium was 33 mg L^−1^; 4*R*-F incorporation led to a nearly identical proinsulin yield, while incorporation of the other two analogs led to a significant decrease in proinsulin expression (7–8 mg L^−1^; Table S2). Proinsulins were refolded, digested with trypsin and carboxypeptidase B, and purified to achieve mature lispro variants (Table S2, and Fig. S1).

**Figure 2:**
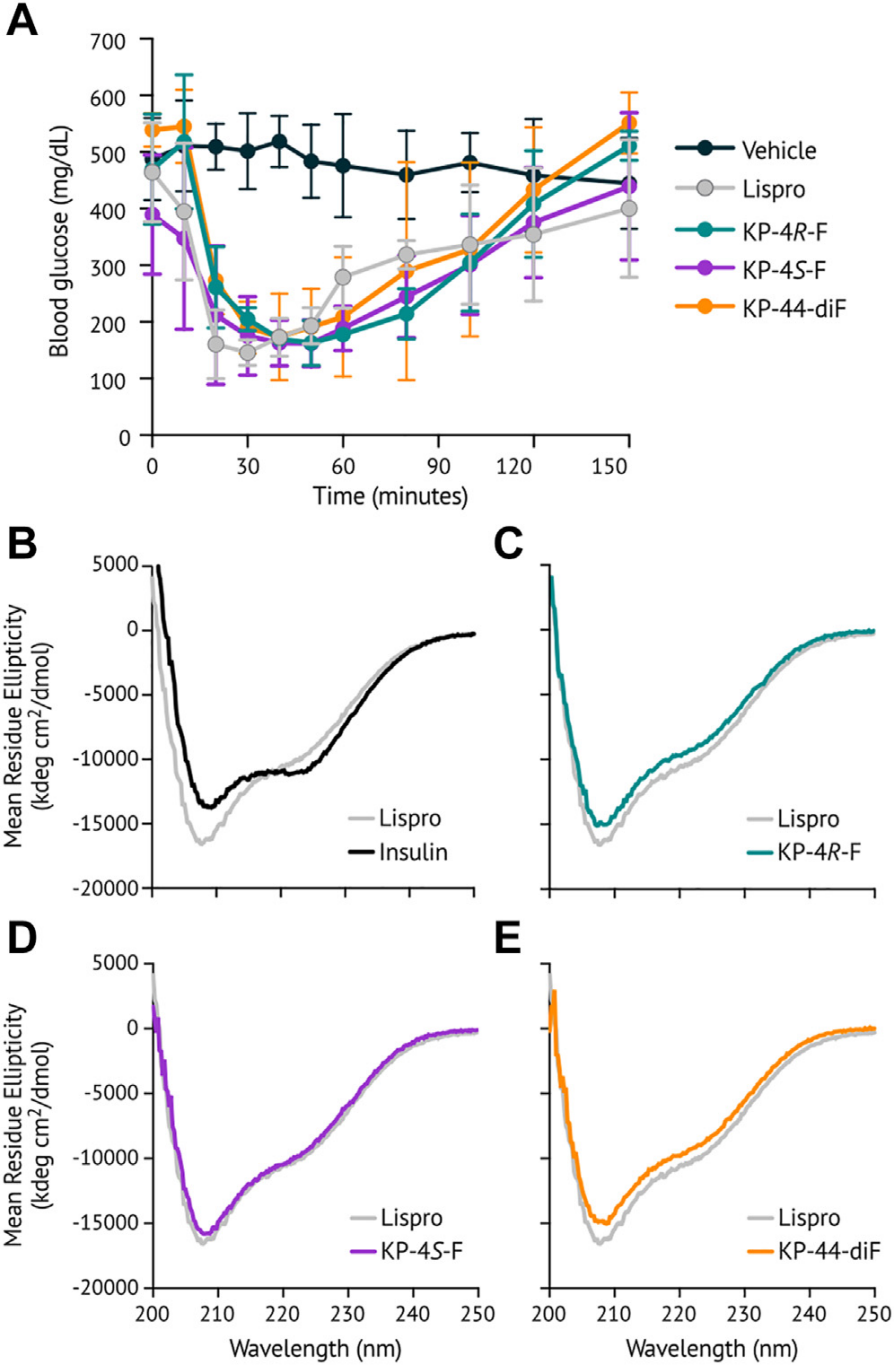
**Biological activity and CD spectroscopy of lispro variants.***A*, lispros were injected subcutaneously into diabetic mice, and blood glucose was measured over time after injection. The data presented here represent the mean ± SD of 3 to 6 biological replicates. *B*–*E*, far-UV circular dichroism spectra (60 μM lispro in 10 mM phosphate buffer, pH 8.0). The spectrum of lispro is overlaid with that of human insulin (*B*), KP-4*R*-F (*C*), KP-4*S*-F (*D*), and KP-44diF (*E*). 4R-F, 4R-fluoroproline; 4S-F, 4S-fluoroproline.

To verify bioactivity, lispro variants were injected subcutaneously into diabetic mice, and blood glucose was monitored over 2.5 h. Mouse models allow determination of activity but not time to onset of action (27). Because the C-terminus of the B-chain does not participate in binding to the insulin receptor (28), we did not expect substitution at position B29 to affect bioactivity. As expected, all lispro variants retained activity (Fig. 2*A*). Further, lower lispro doses—5 to 10% of the standard injection, representing the residual proline-containing lispro present in our lispro analog preparations (Table S1)—did not similarly reduce blood glucose in diabetic mice (Fig. S2). These results demonstrate that our fluorinated variants are active *in vivo*.

The secondary structure of each insulin lispro variant was probed by circular dichroism spectroscopy. Compared to human insulin, which exists as a dimer at 60 μM, the monomeric insulin lispro exhibits increased negative ellipticity at 208 nm and decreased negative ellipticity at 222 nm (Fig. 2*B*), consistent with previous measurements (7). The CD spectra of all of the fluorinated lispro variants were similar to that of lispro (Fig. 2, *C*–*E*), suggesting that fluorination does not alter the secondary structure of the protein. All lispro samples were further analyzed by analytical ultracentrifugation (Fig. S3). To determine the dissociation constant of each variant, we fit the data to a reversible monomer-dimer equilibrium (29). The K_d_ obtained for lispro, 0.65 mM, 95% confidence interval [0.27–1.02], is in reasonable agreement with the value of 1.1 mM previously reported (7). Similar K_d_ values for each fluorinated lispro variant (1.32–1.88 mM, Table S3) confirmed the preference for the monomer state across all samples (Fig. S3, and Table S3).

**Figure 3:**
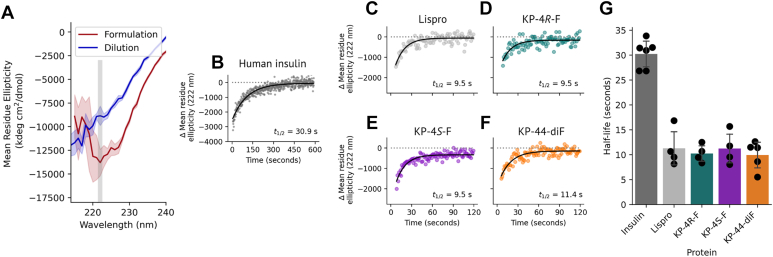
**Hexamer dissociation kinetics of lispro variants.***A*, equilibrium CD spectra of lispro before and after dilution. To measure dissociation kinetics, the decrease in negative ellipticity at 222 nm was monitored over time after dilution. *B*–*F*, representative dissociation kinetics measurements for human insulin (*B*), lispro (*C*), KP-4*R*-F (*D*), KP-4*S*-F (*E*), KP-44diF (*F*). Note the extended x-axis for insulin in panel *B*. G, summary of dissociation half-life values; replicate measurements for each sample originate from at least two separate HPLC fractions, measured on different days. 4R-F, 4R-fluoroproline; 4S-F, 4S-fluoroproline.

We measured the half-life (t_1/2_) for dissociation of the hexamer form of each lispro variant by monitoring the change in negative ellipticity at 222 nm over time after dilution (Figs. 3*A*, S4, and S5). Insulin and lispro variants were formulated to mimic the pharmaceutical formulation (600 μM insulin, 25 mM resorcinol, 250 μM ZnCl_2_); resorcinol was used as the phenolic ligand to slow dissociation (30). Under these conditions, insulin lispro dissociated significantly more rapidly than human insulin (t_1/2_ = 11.3 ± 3.8 and 30.2 ± 2.8 s, respectively), as expected. Monitoring dissociation processes faster than that of lispro is limited by the mixing time of this experiment, though comparisons to human insulin can still be made. All of the fluorinated lispro variants dissociated faster than human insulin under the conditions used here (Fig. 3, *B*–*G*, and Table S4).

**Figure 4:**
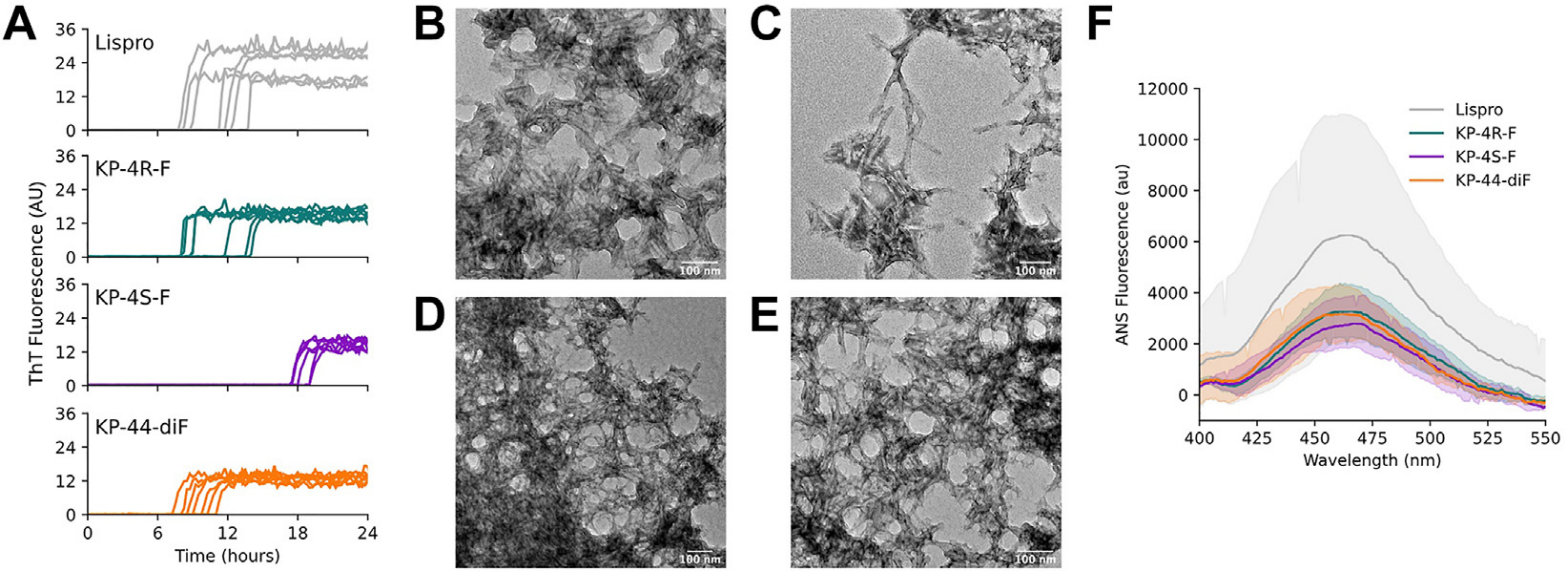
**Fibrillation of lispro variants.***A*, lispro variants (60 μM in 100 mM phosphate buffer, pH 8.0) were incubated at 37 °C with vigorous shaking; fibril formation was monitored by ThT fluorescence. Fibrillation runs were performed on two separate HPLC fractions, each in triplicate or quadruplicate, on two separate days. *B*–*E*, TEM images of lispro (*B*), KP-4*R*-F (*C*), KP-4*S*-F (*D*), and KP-44-diF (*E*) aggregates. *F*, ANS emission spectra of lispro variants (1 μM lispro variant labeled with 5 μM ANS in 100 mM phosphate buffer, pH 8.0). The data presented here indicate the mean ± SD for each variant from measurements of three separate HPLC fractions. 44-diF, 4,4-difluoroproline; 4R-F, 4R-fluoroproline; 4S-F, 4S-fluoroproline; ThT, thioflavin T.

Each insulin lispro variant was subjected to continuous, vigorous shaking at 37 °C to assess its stability against physical denaturation; fibrillation was monitored with the dye thioflavin T, (Fig. 4*A*). Under these conditions, the fibrillation lag time of insulin lispro (10.9 ± 2.2 h) was reduced compared to that of human insulin (16.7 ± 4.1 h) (25), consistent with the conventional view that fibrillation proceeds from the monomer state (11). Replacing ProB29 with 4*R*-F (10.3 ± 2.4 h) or 44-diF (9.0 ± 1.3 h) did not significantly change the fibrillation lag time. Notably, KP-4*S*-F was stabilized against fibril formation (17.9 ± 0.8 h). TEM analysis of the lispro fibrils revealed similar morphology among samples (Fig. 4, *B*–*E*).

We probed the extent of conformational disorder of each lispro variant using the dye 8-anilino-1-naphthalenesulfonic acid (ANS), which exhibits a blue shift in its emission maximum upon binding to hydrophobic patches of proteins indicative of a molten globule state (31). The emission spectra of all lispro variants were similar upon addition of ANS (Fig. 4*F*), suggesting that the observed differences in fibrillation propensity were not due to variations in protein disorder. We note that 4*S*-F more strongly favors the *endo* ring pucker and *cis* amide isomer than the other proline analogs examined in this study (14, 32). Perhaps these conformational preferences at the C terminus of the B-chain inhibit the formation of nuclei that precede fibril formation.

## Discussion

Non-canonical amino acid mutagenesis provides a convenient method for the synthesis of insulin lispro variants fluorinated at ProB29. While most of the properties of the fluorinated variants are similar to those of lispro, we find that 4*S*-fluorination at position ProB29 stabilizes the protein against fibril formation. Approaches to protect insulin against denaturation often rely upon promoting oligomerization (33). However, here we observe stabilization of a monomeric FAI without changes in association behavior. Similar stabilization from the ligand-free, monomer state was observed for an insulin lispro variant that contains a 3-iodo substituent at TyrB26 (34). Iodination slowed hexamer dissociation in *in vitro* studies, suggesting lispro’s rapid-acting behavior might be attenuated after subcutaneous injection in a human. Conversely, we were unable to detect differences in hexamer dissociation kinetics in our fluorinated lispro variants. In neither case is the mechanism of stabilization against fibril formation understood. ncPro substitution can impart long-range conformational effects (35), and the molecular mechanism of insulin fibrillation is not fully established (11). Perhaps the conformational preferences of 4S-F (*endo* ring pucker and *cis* amide isomer) may contribute to delayed fibril formation.

Regardless of the mechanism, the discovery of a monomeric insulin with an extended lag time to fibril formation (KP-4*S*-F) is relevant to the design of improved insulin therapies. Current insulin formulations must be stored at 4 °C and have limited shelf-lives. The inherent sensitivity of insulin to chemical and physical denaturation is especially problematic for long-term storage in continuous subcutaneous insulin infusion pumps (36) and in efforts to provide insulin-replacement therapies to individuals without regular access to refrigeration (37).

The results of this work highlight the ability of non-canonical amino acid mutagenesis to modulate pharmaceutically relevant properties of therapeutic proteins. ncPro mutagenesis allows for the modification of insulin lispro at position B29 without losing the unique conformational restrictions characteristic of proline, and the fluorinated insulin lispro variants introduced here can be produced through straightforward bacterial expression. Further work is needed to elucidate the molecular origins of the stabilization of KP-4*S*-F.

## Experimental procedures

Detailed methods are provided in the Supporting Information.

### Preparation of insulin and lispro variants

Human insulin and lispro variants were prepared as previously described (25); details are provided in the Supporting information. Incorporation efficiencies for all proline analogs exceeded 90% (Table S1).

### Reduction of blood glucose in diabetic mice

Experiments were conducted according to procedures approved by the Institutional Animal Care and Use Committee at the City of Hope. Adult (8–12 week old) male NODscid mice (Jackson Laboratory; stock # 001303) were injected intraperitoneally for three consecutive days with freshly prepared streptozotocin (45 mg kg^−1^ body weight, suspended in 100 mM sodium citrate buffer, pH 4.5); diabetes was confirmed by the detection of high glucose levels (200–600 mg dL^−1^) as measured by a glucomonitor (Freestyle, Abbott Diabetes Care). Lispro analogs were diluted to 100 μg mL^−1^ in formulation buffer (1.6 mg mL^−1^ *m*-cresol, 0.65 mg mL^−1^ phenol, 3.8 mg mL^−1^ sodium phosphate pH 7.4, 16 mg mL^−1^ glycerol, 0.8 μg mL^−1^ ZnCl_2_) and injected (35 μg kg^−1^) subcutaneously at the scruff. Blood glucose was measured from blood sampled from the lateral tail vein.

### Kinetics of hexamer dissociation

Samples were dialyzed overnight against 28.6 mM tris buffer, pH 8.0, then formulated as 600 μM insulin or lispro, 250 μM ZnCl_2_, 25 mM resorcinol, 25 mM tris, pH 8.0. A 20 μl aliquot of the formulation solution was injected into a stirred buffer solution containing 2.98 ml of 25 mM tris, pH 8.0 (150-fold dilution) at 25 °C. Ellipticity was monitored over 120 s at 222 nm. A typical run led to a rapid drop in CD signal as mixing occurred (∼5 s), then a gradual rise to an ellipticity representative of an insulin monomer. Data preceding the timepoint with the greatest negative ellipticity represented the mixing time and were omitted from further analysis. Runs were discarded if the maximum change in mean residue ellipticity from equilibrium did not exceed 750 deg cm^2^ dmol^−1^, which indicated poor mixing. Dissociation data were fit to a mono-exponential function using Scipy (Python). The data presented here are from at least two separate HPLC fractions, measured on different days.

### Analytical ultracentrifugation

Lispro variants (140–206 μM in 100 mM phosphate buffer, pH 8.0) were analyzed by velocity sedimentation at the Canadian Center for Hydrodynamics at the University of Lethbridge using absorbance optics. All samples were measured at 60,000 RPM and 20 °C. Data were analyzed with UltraScan III version 4.0 release 6606 (38). Velocity data were initially fitted with the two-dimensional spectrum analysis (39) to determine meniscus position and time- and radially-invariant noise, and to generate molecular weight distributions. Two-dimensional spectrum analysis results were refined using the genetic algorithm approach (40). Sedimentation and diffusion coefficients derived from the genetic algorithm analysis were transformed to molar mass distributions, assuming a partial specific volume of 0.7248 ml/g for all lispro variants. The enhanced van Holde-Weischet analysis (41) was used to determine diffusion-corrected sedimentation coefficient distributions. For the K_d_ analysis, AUC data were fitted with a discrete model genetic algorithm approach (29), assuming the known molar mass of the monomer and floating the K_d_, k_off_, total concentration, partial specific volumes, and frictional ratios as reported in Table S3.

### Fibrillation lag time

Lispro samples (60 μM in 100 mM sodium phosphate buffer, pH 8.0) were centrifuged (22,000*g*, 1 h, 4 °C); 1 μM thioflavin T was then added. Each lispro variant was shaken continuously in a 96-well, black, clear bottom plate at 37 °C, and fluorescence (444 nm excitation, 485 nm emission) was monitored over time. Fibrillation runs were performed on two separate HPLC fractions, each in triplicate or quadruplicate, on two separate days. The growth phase of each replicate was fit to a linear function; lag times were reported as the x-intercept.

### ANS fluorescence

Lispro variants (1 μM) were mixed with 5 μM ANS in 100 mM phosphate buffer, pH 8.0. Fluorescence emission spectra were measured at ambient temperature (350 nm excitation). Measurements for each lispro were performed in triplicate from three separate HPLC fractions.

## Data availability

The UltraScan software used to analyze the AUC data is open source and freely available from the Github repository (https://github.com/ehb54/ultrascan3). The AUC data is available in openAUC format upon request from Borries Demeler (demeler@gmail.com), and is stored in the UltraScan LIMS server at the Canadian Center for Hydrodynamics.

## Supporting information

This article contains supporting information (23, 24, 25, 29, 38, 39, 40, 41, 42, 43).

## Conflict of interest

David Tirrell is an inventor on U.S. patents that describe the use of non-canonical amino acids in the engineering of insulin and other proteins. The other authors declare that they have no conflicts of interest with the contents of this article.
