# Supplementary material for: 4S-fluorination of ProB29 in insulin lispro slows fibril formation
Source: J Biol Chem. 2024 May 3;300(6):107332. doi: 10.1016/j.jbc.2024.107332 (PMC11154709; doi:10.1016/j.jbc.2024.107332)
Supplement: Supporting information [file mmc1.pdf]

Supporting Information

4S-fluorination of ProB29 in insulin lispro slows fibril formation

Stephanie L. Breunig,[1] Alex M. Chapman,[1] Jeanne LeBon,[2] Janine C. Quijano,[2] Maduni Ranasinghe,[3] Jeffrey Rawson,[2] Borries Demeler,[3,4] Hsun Teresa Ku,[2,5] and David A. Tirrell[1*]

[1]Division of Chemistry and Chemical Engineering, California Institute of Technology, Pasadena, California 91125, United States.
[2]Department of Translational Research and Cellular Therapeutics, Arthur Riggs Diabetes and Metabolism Research Institute, Beckman Research Institute City of Hope, Duarte, California 91010, United States.
[3]Department of Chemistry and Biochemistry, University of Lethbridge, Lethbridge, Alberta T1K 3M4, Canada.
[4]Department of Chemistry and Biochemistry, University of Montana, Missoula, Montana 59812, United States
[5]Irell & Manella Graduate School of Biological Science, City of Hope, Duarte, California 91010, United States.

[*]Corresponding author. Email: tirrell@caltech.edu

Table of Contents

Materials and methods S2
- Chemicals S2
- Enzymes S2
- Strains and plasmids S2
- Primers S2
- Nucleotide and amino acid sequences S3
- Proinsulin expression S4
- Proinsulin refolding S5
- Lispro maturation and purification S5
- Preparation of lispro variants for mouse experiments S6
- MALDI-TOF MS S6
- Reduction of blood glucose in diabetic mice S6
- Circular dichroism spectroscopy S7
- Analytical ultracentrifugation S7
- Fibrillation S8
- Transmission electron microscopy S8
- ANS fluorescence S8

Table S1: Mass spectrometry characterization of lispro variants S9
Table S2: Lispro yields S10
Table S3: Summary of lispro variant hydrodynamic and thermodynamic characterization by analytical ultracentrifugation S11
Table S4: Summary of lispro variant characterization S12
Figure S1: Purity of lispro samples assessed by SDS-PAGE S13

Figure S2: Effects of varying doses of lispro on the blood glucose levels in diabetic mice S14
Figure S3: Diffusion-corrected van Holde-Weischet integral sedimentation coefficient distributions.....S15
Figure S4: Changes in CD signal after dilution are not due to protein denaturation..............................S16
Figure S5: Equilibrium CD spectra after dilution S17
References...S18

Materials and methods

Chemicals:

All chemicals were purchased from MilliporeSigma unless otherwise indicated. 4*R*-fluoroproline (4*R*-F) and 4*S*-fluoroproline (4*S*-F) were purchased from Bachem. 4,4-difluoroproline (44-diF) was purchased from Synthonix. All proline analogs were used as received.

Enzymes:

Gibson Assembly enzymes were purchased as the Repliqa HiFi assembly mix from Quantabio. Trypsin was purchased from MilliporeSigma. Carboxypeptidase B was purchased from Worthington Biochemical. Glu-C peptidase was purchased from Promega.

Strains and plasmids:

The proline-auxotrophic *E. coli* strain CAG18515 was obtained from the Coli Genetic Stock Center (CGSC) at Yale University. Strain DH10B was used for all cloning applications.

The plasmid pQE80_H27R-PI-KP_proS contains an IPTG-inducible proinsulin-lispro gene and an *E. coli* prolyl-tRNA synthetase gene controlled by its endogenous promoter. Proinsulin is translationally fused to an N-terminal leader peptide (H27R) that increases expression yields,(42) and a 10x-his tag to facilitate proinsulin enrichment after refolding. The ProB28-LysB29 inversion present in insulin lispro was installed in plasmid pQE80_H27R-PI_proS.(25) We used a two-part Gibson Assembly approach with the two sets of primers described below. The two overlap regions were at the site of mutation, and within the ampicillin resistance gene selection marker. Correct installation of the desired mutation was verified by Sanger sequencing.

Primers:

DNA oligos were purchased from Integrated DNA Technologies (IDT). Nucleotides responsible for the proline-lysine inversion are underlined.

Gibson assembly fragment 1:

SLB3099_GA-fwd: CGTGGTTTCTTCTACACGAAACCGACCCGCCGTGAAG
AmpR-GA_rev: GACAGTAAGAGAATTATGCAGTG

Gibson assembly fragment 2:

AmpR-GA_fwd: GCAGTGTTATCACTCATGG
SLB3099_GA-rev: CAGCTTCACGGCGGGTCGGTTTCGTGTAGAAGAAACCACG

Nucleotide and amino acid sequences:

<u>H27R-PI-KP</u>: The mutations responsible for the proline-lysine inversion are <u>underlined</u>.

ATGACAATGATCACTAATTCACCCGAGATTTCCCACCATCATCATCATCATCACCACCACCATCAGTTGAT
CTCGGAGGCCCGTTTTGTGAACCAGCACCTGTGCGGTAGCCACCTGGTGGAAGCTCTGTACCTGGTTTG
CGGTGAGCGTGGTTTCTTCTACACG<u>AAA</u>A<u>CC</u>GACCCGCCGTGAAGCTGAAGATCTGCAGGTGGGCCAGG
TAGAACTGGGCGGTGGTCCGGGTGCCGGCTCTCTGCAACCGCTGGCACTGGAAGGTTCCCTGCAAGCG
CGTGGTATCGTAGAGCAGTGCTGTACTTCTATCTGCTCCCTGTACCAGCTGGAGAACTACTGTAATTAA

The sequence of the H27R leader peptide is underlined, proinsulin is in **bold**. The A-chain and B-chain in mature insulin are colored **red** and **blue**, respectively.

<u>MTMITNSPEISHHHHHHHHHHQLISEAR</u>**FVNQHLCGSHLVEALYLVCGERGFFYTKPTRREAEDLQVGQVE
LGGGPGAGSLQPLALEGSLQARGIVEQCCTSICSLYQLENYCN**

<u>proS</u>: the endogenous *proS* promoter is <u>underlined</u>, the coding sequence is in UPPERCASE.

attcacgc<u>ccttctcttttgacatttcttttgcactggtaaactaaatcac</u>tttttttgtcccaggctcgccttgagcctgttctaccttccaac
tggaaccgtaacaacATGCGTACTAGCCAATACCTGCTCTCCACTCTCAAGGAGACACCTGCCGACGCCGAG
GTGATCAGCCATCAGCTGATGCTGCGCGCCGGGATGATCCGCAAGCTGGCCTCCGGGTTATATACCTGG
CTGCCGACCGGCGTGCGCGTTCTGAAAAAAGTCGAAAACATCGTGCGTGAAGAGATGAACAACGCCGG
TGCGATCGAGGTGTCGATGCCGGTGGTTCAGCCAGCCGATTTGTGGCAAGAGAGTGGTCGTTGGGAAC
AGTACGGTCCGGAACTGCTGCGTTTTGTTGACCGTGGCGAGCGTCCGTTCGTACTCGGCCCAACTCATG
AAGAAGTTATCACTGACCTGATTCGTAACGAGCTTAGCTCTTACAAACAGCTGCCGCTGAACTTCTATCA
GATCCAGACCAAGTTCCGCGACGAAGTGCGTCCGCGTTTCGGCGTCATGCGTTCCCGCGAATTCCTGAT
GAAAGATGCTTACTCTTTCCATACTTCTCAGGAATCCCTGCAGGAAACCTACGATGCAATGTATGCGGCC
TACAGCAAAATCTTCAGCCGCATGGGGCTGGATTTCCGCGCCGTACAAGCCGACACCGGTTCTATCGGC
GGCAGCGCCTCTCACGAATTCCAGGTGCTGGCGCAGAGCGGTGAAGACGATGTGGTCTTCTCCGACACC
TCTGACTATGCAGCGAACATTGAACTGGCAGAAGCTATCGCGCCGAAAGAACCGCGCGCTGCTGCTACC
CAGGAAATGACGCTGGTTGATACGCCGAACGCGAAAACCATCGCGGAACTGGTTGAACAGTTCAATCT
GCCGATTGAGAAAACGGTTAAGACTCTGCTGGTTAAAGCGGTTGAAGGCAGCAGCTTCCCGCAGGTTG
CGCTGCTGGTGCGCGGTGATCACGAGCTGAACGAAGTTAAAGCAGAAAAACTGCCGCAGGTTGCAAGC
CCGCTGACTTTCGCGACCGAAGAAGAAATTCGTGCCGTGGTTAAAGCCGGTCCGGGTTCACTGGGTCCG
GTAAACATGCCGATTCCGGTGGTGATTGACCGTACCGTTGCGGCGATGAGTGATTTCGCTGCTGGTGCT
AACATCGATGGTAAACACTACTTCGGCATCAACTGGGATCGCGATGTCGCTACCCCGGAAGTTGCAGAT
ATCCGTAACGTGGTGGCTGGCGATCCAAGCCCGGATGGCCAGGGTAGGCTGCTGATCAAACGTGGTAT
CGAAGTTGGTCACATCTTCCAGCTGGGTACCAAGTACTCCGAAGCACTGAAAGCCTCCGTACAGGGTGA
AGATGGCCGTAACCAAATCCTGACGATGGGTTGCTACGGTATCGGGGTAACGCGTGTGGTAGCTGCGG
CGATTGAGCAGAACTACGACGAACGAGGCATCGTATGGCCTGACGCTATCGCGCCGTTCCAGGTGGCG
ATTCTGCCGATGAACATGCACAAATCCTTCCGCGTACAAGAGCTTGCTGAGAAACTGTACAGCGAACTG
CGTGCACAAGGTATCGAAGTGCTGCTGGATGACCGCAAAGAGCGTCCGGGCGTGATGTTTGCTGATAT
GGAACTGATCGGTATTCCGCACACTATTGTGCTGGGCGACCGTAACCTCGACAACGACGATATCGAATA
TAAATATCGTCGCAACGGCGAGAAACAGTTAATTAAGACTGGTGACATCGTCGAATATCTGGTGAAACA
GATTAAAGGCTGA

MRTSQYLLSTLKETPADAEVISHQLMLRAGMIRKLASGLYTWLPTGVRVLKKVENIVREEMNNAGAIEVSMP
VVQPADLWQESGRWEQYGPELLRFVDRGERPFVLGPTHEEVITDLIRNELSSYKQLPLNFYQIQTKFRDEVRP
RFGVMRSREFLMKDAYSFHTSQESLQETYDAMYAAYSKIFSRMGLDFRAVQADTGSIGGSASHEFQVLAQS
GEDDVVFSDTSDYAANIELAEAIAPKEPRAAATQEMTLVDTPNAKTIAELVEQFNLPIEKTVKTLLVKAVEGSS
FPQVALLVRGDHELNEVKAEKLPQVASPLTFATEEEIRAVVKAGPGSLGPVNMPIPVVIDRTVAAMSDFAAG
ANIDGKHYFGINWDRDVATPEVADIRNVVAGDPSPDGQGRLLIKRGIEVGHIFQLGTKYSEALKASVQGEDG
RNQILTMGCYGIGVTRVVAAAIEQNYDERGIVWPDAIAPFQVAILPMNMHKSFRVQELAEKLYSELRAQGIE
VLLDDRKERPGVMFADMELIGIPHTIVLGDRNLDNDDIEYKYRRNGEKQLIKTGDIVEYLVKQIKG

Proinsulin expression:

All lispro variants were expressed as the corresponding proinsulins using strain CAG18515/pQE80_H27R-PI-KP_proS. This is a proline auxotrophic strain of *E. coli* which carries a plasmid for prolyl-tRNA synthetase over-expression and inducible expression of proinsulin-lispro.

A single colony was used to inoculate 70 mL of Luria Bertani (LB) medium containing ampicillin, and the culture was grown to stationary phase overnight at 37°C. The overnight culture was used to inoculate 5 L (as 4x1.25 L cultures) of 1x Andrew’s Magical Medium (AMM) (43) , a defined medium that contains all 20 proteinogenic amino acids, in 2.8 L Fernbach flasks. The composition of AMM was the following: 3.60 g L^{-1} glucose, 3.5 g L^{-1} KH_2PO_4, 6.56 g L^{-1} K_2HPO_4•$3H_2O$, 3.5 g L^{-1} $(NH_4)_2HPO_4$, 8.37 g L^{-1} MOPS, 0.72 g L^{-1} tricine, 2.92 g L^{-1} NaCl, 0.51 g L^{-1} NH_4Cl, 0.26 g L^{-1} $MgCl_2$•$7H_2O$, 50 mg L^{-1} K_2SO_4, 0.246 mg L^{-1} $MgSO_4$•$7H_2O$, 12.3 mg L^{-2} $CaCl_2$,•$2H_2O$, 2.8 mg L^{-1} $FeSO_4$•$7H_2O$, 0.5 mg L^{-1} thiamine, 24 µg L^{-1} boric acid, 1 µg L^{-1} trace metals (Cu^{2+}, Mn^{2+}, Zn^{2+}, MoO_4^{2-}), and 50 mg L^{-1} each amino acid.

When growth reached mid-exponential phase (OD_{600} ~0.8), a medium shift was performed: cells were pelleted by centrifugation (5 kg, 5 min, 4°C) and washed twice with ice-cold 0.9% NaCl. Cells were resuspended in 1 L of 1.25x AMM -Pro, a 1.25x concentrated form of AMM that omits proline. To deplete residual proline, cells were incubated for 30 min at 37°C. A 250 mL volume of a solution containing 2.5 mM ncPro and 1.5 M NaCl was then added (0.5 mM ncPro and 0.3 M NaCl working concentrations). For the incorporation of 44-diF, the concentration of NaCl was 2.5 M (0.5 M working concentration). After 30 min of incubation at 37°C for ncPro uptake, proinsulin expression was induced by the addition of isopropylthio-β-galactosidase (IPTG, 1 mM). Cultures were incubated overnight at 37°C; cells were then harvested by centrifugation and stored at -80°C until further processing.

Proline-containing proinsulins were expressed in rich medium: proinsulin-lispro was expressed using strain CAG18515 harboring plasmid pQE80-H27R-PI-KP_proS in 6 L (as 6 x 1.0 L cultures) of Terrific Broth (TB). IPTG (1 mM) was added at mid-log phase (OD_{600} ~0.8) to induce proinsulin expression. Cultures were incubated at 37°C for 3 h, after which cells were harvested by centrifugation and stored at -80°C until further processing.

Proinsulin refolding:

Cell pellets were warmed from -80°C to room temperature, then resuspended in 5 mL IB buffer (50 mM tris, 100 mM NaCl, 1 mM EDTA, pH 8.0) per gram cell pellet. Lysozyme (1 mg L^{-1}) and phenylmethylsulfonyl fluoride (PMSF, 1 mM) were added, and the slurry was placed on ice for 30 min. Cells were lysed by sonication, the lysate was centrifuged (14,000 g, 30 min, 4°C), and the soluble fraction was discarded. The pellet was washed twice with IB buffer + 1% Triton X-100, once with IB buffer, and once with water; this final step required extended centrifugation (14,000 g, 45 min). A minimal amount of water was used to resuspend the washed inclusion body pellet, and the mass of proinsulin in the inclusion body pellet was estimated by SDS-PAGE.

To prepare for proinsulin refolding, the proinsulin concentration was adjusted to 1 mg proinsulin per L total slurry by resuspending the inclusion body in 3 M urea and 10 mM cysteine in water. The pH was adjusted to 12 and sample stirred for 1 h at room temperature to dissolve proinsulin. At this stage, ncPro incorporation was assessed by MALDI-TOF, as described in the section entitled “MALDI-TOF MS” below. The solubilized proinsulin solution was diluted ten-fold into refolding buffer (10 mM N-cyclohexyl-3-aminopropanesulfonic acid, CAPS; pH 10.6) that had been pre-cooled to 4°C. The pH of the refolding solution was adjusted to 10.7 and the sample stored at 4°C; throughout the refolding process, the solution pH was periodically adjusted so that it remained between 10.6 and 10.8. Proinsulin refolding progress was monitored by reverse-phase HPLC, and usually reached completion within 50 h.

Proinsulin was enriched from the refolding solution after adjusting the pH to 8.0 and incubating the sample overnight with Ni-NTA resin and 10 mM imidazole. The resin was washed with wash buffer (25 mM imidazole in PBS, pH 8.0), and proinsulin was eluted with elution buffer (250 mM imidazole in PBS, pH 8.0). Fractions that contained proinsulin were combined and dialyzed extensively against 10 mM sodium phosphate, pH 8.0.

Lispro maturation and purification:

Refolded and dialyzed proinsulin was warmed to 37°C and digested with trypsin (20 U mL^{-1}) and carboxypeptidase-B (10 U mL^{-1}) at 37°C for 2.5 h to remove the N-terminal tag and C-chain. The pH was adjusted to ~3 with 6 N HCl to halt digestion.

Lispro variants were immediately purified after proteolysis by reverse-phase HPLC on a C_4 column (Penomenex Jupiter 5 µm particle size, 300 Å pore size, 250x10 mm) using 0.1% trifluoroacetic acid (TFA) in water (solvent A) and 0.1% TFA in acetonitrile (solvent B) as mobile phases. A gradient of 25-32% solvent B was applied over 65 min, and fractions that contained lispro were collected. Aliquots were removed at this stage for purity analysis; the remaining portion of each lispro-containing fraction was lyophilized. Each fraction was analyzed by analytical reverse-phase HPLC, MALDI-TOF MS (Figure 1h-k), and SDS-PAGE (Figure S1) to verify sample quality and ensure ≥95% purity for all downstream analyses. Lyophilized powders were stored at -20°C until further use.

Preparation of lispro variants for mouse experiments:

We used a previously-developed protocol to express lispro variants used in mouse studies.(23, 24) Briefly, the hexahistidine-tagged version of proinsulin (lacking the H27R leader peptide) was expressed in M9 medium using the strain CAG18515 harboring plasmid pQE80PI-proS. The medium shift described above was used to incorporate the ncPro residue of interest, and proinsulin was expressed for 2.5 h. After cell lysis, proinsulin was solubilized with 8 M urea and isolated from the washed inclusion body fraction by Ni-NTA under denaturing conditions. Refolding was achieved after oxidative sulfitolysis by gentle agitation at 12°C overnight in 0.5 mM BME, 0.3 M urea, 50 mM glycine, pH 12. After dialysis, lyophilization, and proteolysis by trypsin and carboxypeptidase B, mature lispro variants were purified by reverse-phase HPLC.

During the course of this work, we found that the refolding protocol described earlier resulted in an approximately seven-fold increase in refolding yield, and higher purity samples.

MALDI-TOF MS:

To assess levels of incorporation of ncPros into the corresponding proinsulins, samples were subjected to Glu-C digestion, which yields a peptide fragment that contains ProB29 (^{50}RGFFYTK**P**TRRE). A 20 µL aliquot of the solubilized proinsulin-containing inclusion body fraction was subjected to cysteine reduction (5 mM dithiothreitol, DTT; 55°C for 20 min) and alkylation (15 mM iodoacetamide, room temperature for 15 min in the dark), then diluted 10-fold into 100 mM NH_4HCO_3, pH 8.0 (100 µL final volume). Samples were digested with 0.6 µL Glu-C (0.5 µg μL^{-1} in ddH_20) at 37°C for 2.5 h. The digestion reaction was quenched by adding 10 µL of 5% TFA. Peptides were desalted using ZipTip C_{18} columns (MilliporeSigma) according to the manufacturer's protocol. Desalted peptides (in 50% acetonitrile, ACN; 0.1% TFA) were diluted 3:1 into the matrix solution (α-cyanohydroxycinnamic acid in 50% ACN, 0.1% TFA) and analyzed by MALDI-TOF MS. Analog incorporation was calculated by comparing the area under the curve (AUC) of the ncPro form of the peptide (m/z = 1576 for 4*R*-F and 4*S*-F, and 1595 for 44-diF) with the AUC of the canonical proline peptide (m/z = 1558).

HPLC-purified insulin and lispros were analyzed as full-length, mature proteins. Aliquots directly from HPLC purification (~30% ACN, 0.1% TFA) were mixed 1:1 with matrix solution (sinapic acid in 30% ACN, 0.1% TFA) before being analyzed by MALDI-TOF MS.

Reduction of blood glucose in diabetic mice:

NODscid mice (NOD.CB17-Prkdc^{scid}/J; RRID: IMSR_JAX:001303) were obtained from Jax Mice (Bar Harbor, ME). Mice were maintained under specific pathogen-free conditions, and experiments were conducted according to procedures approved by the Institutional Animal Care and Use Committee (IACUC) at the City of Hope. Adult (8-12 week old) male NODscid mice were injected intraperitoneally (45 mg kg^{-1} day^{-1} for 3 consecutive days) with freshly prepared streptozotocin (STZ) in 50 mM citrate buffer, pH 4.5 to induce diabetes. Diabetes was confirmed 3 weeks after the last dose of STZ by detection of high glucose levels (200-600 mg dL^{-1}) as measured by a glucomonitor (Freestyle, Abbott Diabetes Care, Alameda, CA) in blood sampled from the lateral

tail vein. Lispro analogs were diluted to 100 µg mL^{-1} in formulation buffer (1.6 mg mL^{-1} m-cresol, 0.65 mg mL^{-1} phenol, 3.8 mg mL^{-1} sodium phosphate pH 7.4, 16 mg mL^{-1} glycerol, 0.8 µg mL^{-1} $ZnCl_2$). Insulin analogs were injected (35 µg kg^{-1}) subcutaneously at the scruff and blood glucose was measured at 0, 10, 20, 30, 40, 50, 60, 80, 100, 120, and 150 min between 1400 to 1700 military time.

To determine the effect of lower lispro doses on blood glucose concentration, commercial insulin lispro (Humalog, Eli Lilly) was diluted in formulation buffer and injected subcutaneously into STZ-induced diabetic NODscid mice (15-18 week old male). Lispro doses were as follows: 35 mg kg^{-1} (100%), 3.5 mg kg^{-1} (10%), and 1.75 mg kg^{-1} (5%), and blood glucose was measured with a OneTouch Verio blood glucose meter.

Circular dichroism spectroscopy:

Equilibrium measurements: The circular dichroism spectra of insulin or lispro samples (60 µM in 100 mM sodium phosphate, pH 8.0) were measured at 25°C in 1 mm quartz cuvettes using a step size of 0.5 nm and averaging time of 1 s on an Aviv Model 430 Circular Dichroism Spectrophotometer. A reference buffer spectrum was subtracted from each sample spectrum.

Kinetic measurements: Insulin or lispro samples in 100 mM sodium phosphate buffer pH 8.0 were dialyzed overnight against 28.6 mM tris buffer, pH 8.0 (Slide-A-Lyzer dialysis cassettes, 3.5 kDa MWCO, ThermoFisher). Samples were formulated as follows: 600 µM lispro, 250 µM $ZnCl_2$, 25 mM resorcinol, 25 mM tris buffer, pH 8. A 20 µL volume of the insulin formulation was injected into a stirred buffer solution containing 2.98 mL of 25 mM tris, pH 8.0 in a 10 mm quartz cuvette (150-fold dilution). Ellipticity was monitored at 222 nm over 120 s (1 s kinetic interval, 0.5 s time constant) at 25°C. A typical run led to a rapid drop in CD signal as mixing occurred (~5 s), then a gradual rise to an equilibrium ellipticity representative of an insulin monomer. Data preceding the timepoint with the greatest negative ellipticity represented the mixing time; these data were omitted from further analysis. Runs were discarded if the maximum change in mean residue ellipticity from equilibrium did not exceed 750 deg cm^2 $dmol^{-1}$, which indicated poor mixing. The remaining data were fit to a mono-exponential function using Scipy (Python); data presented here are from at least two separate HPLC fractions, measured on two different days.

An equilibrium spectrum for each protein was obtained after dilution; all spectra were indicative of an insulin monomer (Figure S3). The CD spectrum of lispro under pre-dilution formulation conditions was obtained using a 0.1 mm quartz cuvette. A blank spectrum containing all buffers and ligands was subtracted from the sample spectrum.

Analytical ultracentrifugation:

Lispro variants were formulated at 140-206 µM in 100 mM phosphate buffer, pH 8.0. Velocity sedimentation experiments were performed at the Canadian Center for Hydrodynamics at the University of Lethbridge using absorbance optics. High concentrations samples were measured at 237 nm, intermediate concentration samples at 232 nm, and low concentration samples at 225 nm. All samples were measured at 60,000 RPM and 20°C in standard Beckman Coulter cell

housings fitted with 0.3 cm epon-charcoal centerpieces for the high concentration samples, and 1.2 cm epon-charcoal centerpieces for the intermediate and low concentration samples. All cells were fitted with sapphire windows. Data were analyzed with UltraScan III version 4.0 release 6606. (38) Velocity data were initially fitted with the two-dimensional spectrum analysis(39) to determine meniscus position and time- and radially-invariant noise, and to generate molecular weight distributions. 2DSA results were refined using the genetic algorithm (GA) approach.(40) Sedimentation and diffusion coefficients derived from the GA analysis were transformed to molar mass distributions (see Figure S2), assuming a partial specific volume of 0.7248 ml/g for all lispro variants. The enhanced van Holde-Weischet analysis(41) was used to determine diffusion-corrected sedimentation coefficient distributions. For the K_d analysis, AUC data were fitted with a discrete model genetic algorithm (DMGA) approach,(29) assuming the known molar mass of the monomer, and floating the K_d, k_{off}, total concentration, partial specific volumes, and frictional ratios as reported in Table S3.

Fibrillation:

Lispro samples (60 µM in 100 mM sodium phosphate, pH 8.0) were centrifuged at 22,000 g for 1 h at 4°C, before 1 µM thioflavin T (ThT) was added. Each lispro (200 µL) was added to a 96-well, black, clear bottom plate (Greiner Bio-One) and sealed. Samples were shaken continuously at 960 rpm on a Varioskan multimode plate reader at 37°C, and fluorescence readings were recorded every 15 min (444 nm excitation, 485 nm emission). Fibrillation runs were performed on at least two separate HPLC fractions, each in triplicate or quadruplicate, and on two different days. The growth phase of each fibrillation replicate was fit to a linear function; fibrillation lag times were reported as the x-intercept of this fit. Fibril samples were stored at 4°C until analysis by transmission electron microscopy (TEM).

Transmission electron microscopy:

Lispro fibrils were briefly centrifuged (5 kg, 1 min), then washed twice and resuspended in ddH_2O. Fibrils were stained with 2% uranyl acetate on a 300-mesh formvar/carbon coated copper grid (Electron Microscopy Sciences) and imaged on a Tecnai T12 LaB6 120 eV transmission electron microscope.

ANS fluorescence:

Lispro variants (1 µM) were mixed with 5 µM ANS in 100 mM phosphate buffer, pH 8.0. Fluorescence emission spectra were measured in 1 cm quartz cuvettes at ambient temperature using a PTI QuantaMaster fluorescence spectrofluorometer. A 2 nm s^{-1} scan rate and 350 nm excitation wavelength were used. Measurements for each variant were performed in triplicate from three separate HPLC fractions.

Table S1. Mass spectrometry characterization of lispro variants.

	Digested peptide			Mature insulin	
Protein	Expected m/z	Observed m/z	Incorporation efficiency	Expected m/z	Observed m/z
Lispro	1557.78	1558.0	–	5808.6	5807.7 ± 0.4
KP-4*R*-F	1575.82	1575.737 ± 0.008	0.961 ± 0.005	5826.6	5826.31 ± 0.03
KP-4*S*-F	1575.82	1575.53 ± 0.01	0.948 ± 0.033	5826.6	5826.5 ± 0.1
KP-44-diF	1593.81	1593.57 ± 0.01	0.912 ± 0.016	5844.6	5844.1 ± 0.7

Table S2. Lispro yields.

Protein	Proinsulin yield (mg L^{-1})‡	Approx. insulin yield (mg L^{-1})
Lispro*	33	n.d.#
KP-4*R*-F	32	3.4
KP-4*S*-F	7	2.2
KP-44-diF	8	2.4

‡Yields determined by measuring absorbance (280 nm) after Ni-NTA enrichment following proinsulin refolding.
*Expressed in terrific broth (TB)
#n.d., not determined

Table S3. Summary of lispro variant hydrodynamic and thermodynamic characterization by analytical ultracentrifugation. Values in brackets represent the 95% confidence interval. s, sedimentation coefficient; D, diffusion coefficient; PSV, partial specific volume.

	Monomer			Dimer				
Sample	s (S)	D ($\mu m^2/s$)	Frictional ratio	s (S)	D ($\mu m^2/s$)	Frictional ratio	PSV (ml g^{-1})	K_d (mM)
WT-KP	0.85 [0.81, 0.88]	1.26 [1.16, 1.35]	1.44 [1.32, 1.57]	1.48 [1.25, 1.72]	1.10 [0.99, 1.21]	1.31 [1.20, 1.43]	0.72 [0.70, 0.74]	0.65 [0.27, 1.02]
KP-4RF	0.90 [0.88, 0.93]	1.20 [1.14, 1.25]	1.54 [1.47, 1.61]	1.99 [1.58, 2.39]	1.31 [1.08, 1.55]	1.12 [0.90, 1.35]	0.69 [0.69, 0.70]	1.88 [0.97, 2.79]
KP-4SF	1.03 [1.00, 1.05]	1.32 [1.29, 1.36]	1.40 [1.35, 1.44]	1.87 [1.63, 2.10]	1.20 [1.05, 1.35]	1.23 [1.08, 1.37]	0.68 [0.67, 0.69]	1.58 [0.66, 2.50]
KP-44DIF	1.00 [0.95, 1.04]	1.24 [1.16, 1.32]	1.50 [1.40, 1.60]	1.59 [1.30, 1.88]	0.99 [0.84, 1.14]	1.50 [1.40, 1.60]	0.67 [0.64, 0.70]	1.32 [0.0, 2.80]

Table S4. Summary of lispro variant characterization.

Protein	Ellipticity ratio (208/222 nm)[‡]	Hexamer dissociation $t_{1/2}$ (s)	Fibrillation lag time (h)	ANS emission maximum (nm)
Lispro	1.63 ± 0.07	11.3 ± 3.8	10.9 ± 2.2	466 ± 2
KP-4*R*-F	1.62 ± 0.06	10.2 ± 1.6	10.3 ± 2.4	464 ± 4
KP-4*S*-F	1.59 ± 0.06	11.2 ± 3.3	17.9 ± 0.8	463 ± 6
KP-44-diF	1.61 ± 0.05	9.9 ± 2.9	9.0 ± 1.3	462 ± 5
Insulin	1.24 ± 0.03[*]	30.2 ± 2.8	16.6 ± 4.1[*]	470 ± 5[*]

[‡]60 µM insulin or lispro variant; 100 mM phosphate, pH 8.0

[*]Ref (25)

Figure S1. Purity of lispro samples assessed by SDS-PAGE. HPLC-purified lispro (a), KP-4*R*-F (b), KP-4*S*-F (c), and KP-44diF (d) were analyzed by SDS-PAGE to validate purity; shown are representative lanes corresponding to individual HPLC fractions.

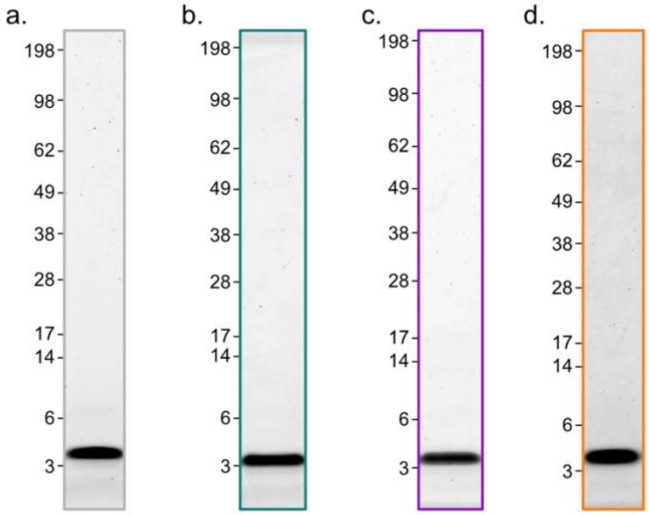

Figure S2. Effects of varying doses of lispro on the blood glucose levels in diabetic mice. Commercial lispro (Humalog, Eli Lilly) was injected subcutaneously in diabetic mice at 5, 10, and 100% of the standard dose (35 mg kg^{-1}), and blood glucose was measured over time. The data presented here represent the mean ± standard deviation of 4 biological replicates.

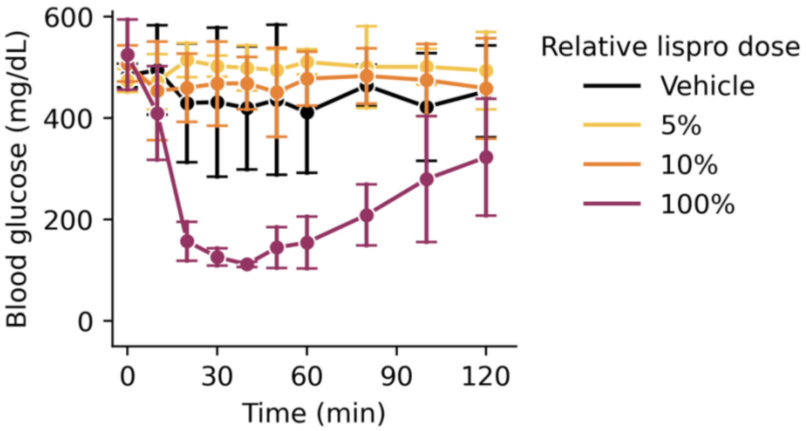

Figure S3. Diffusion-corrected van Holde – Weischet integral sedimentation coefficient distributions. Lispro variants were formulated in 100 mM phosphate, pH 8.0 at the indicated concentrations, and analyzed by sedimentation velocity analytical ultracentrifugation.

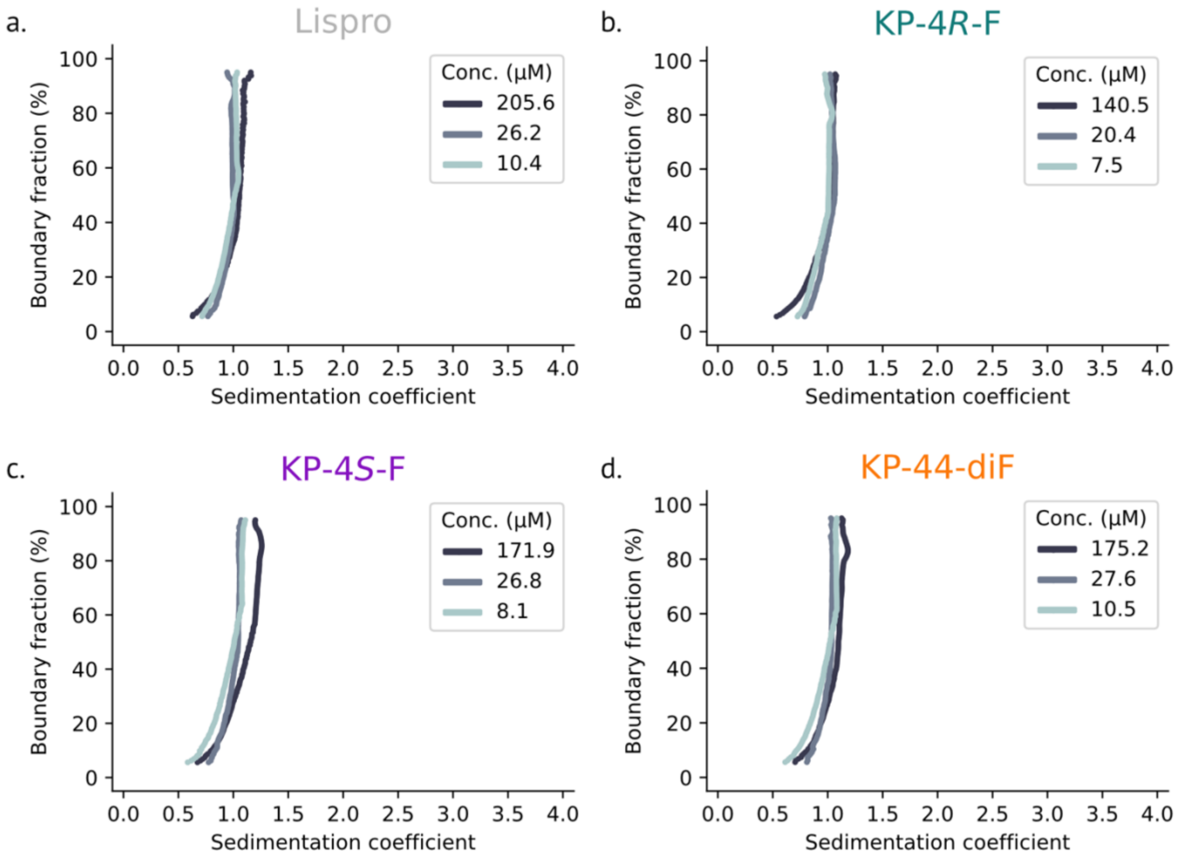

Figure S4. Changes in CD signal after dilution are not due to protein denaturation. At 60 µM, human insulin is expected to exist as a dimer at pH 8, as a monomer in 20% ethanol, and in denatured form in 8 M guanidinium chloride. These spectra are overlaid with equilibrium spectra collected before and after lispro dilution for kinetic CD measurements. Spectra below 210-215 nm were omitted for some samples due to high levels of buffer absorbance at these wavelengths.

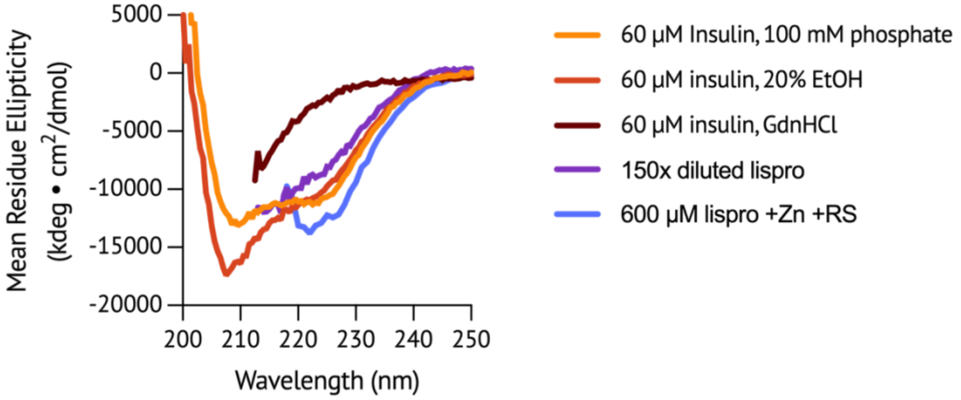

Figure S5. Equilibrium CD spectra after dilution. Equilibrium far-UV spectra of lispro (a), KP-4*R*-F (b), KP-4*S*-F (c), and KP-44diF (d) after 150-fold dilution from the hexamer formulation. Conditions after dilution from the hexamer formulation are as follows: 4 µM lispro variant, 167 µM resorcinol, 1.67 µM $ZnCl_2$, 25 mM tris buffer, pH 8.0. Resorcinol absorbance interferes with CD signal at shorter wavelengths (~205 nm) under these conditions.

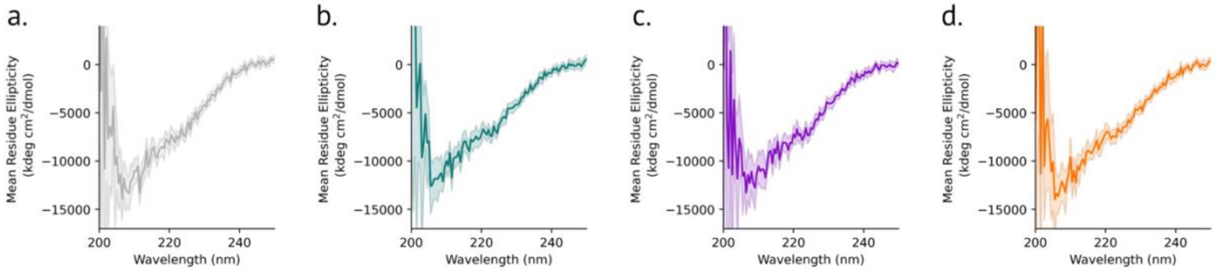

References

23. Fang, K. Y., Lieblich, S. A., and Tirrell, D. A. (2019) Replacement of ProB28 by pipecolic acid protects insulin against fibrillation and slows hexamer dissociation. *J Polym Sci A Polym Chem*. **57**, 264–267
24. Lieblich, S. A., Fang, K. Y., Cahn, J. K. B., Rawson, J., LeBon, J., Teresa Ku, H., and Tirrell, D. A. (2017) 4S-Hydroxylation of Insulin at ProB28 Accelerates Hexamer Dissociation and Delays Fibrillation. *J. Am. Chem. Soc.* **139**, 8384–8387
25. Breunig, S. L., Quijano, J. C., Donohue, C., Henrickson, A., Demeler, B., Ku, H. T., and Tirrell, D. A. (2023) Incorporation of aliphatic proline residues into recombinantly-produced insulin. *ACS Chem. Biol.* **18**, 2574-2581
29. Demeler, B., Brookes, E., Wang, R., Schirf, V., and Kim, C. A. (2010) Characterization of Reversible Associations by Sedimentation Velocity with UltraScan. *Macromol Biosci*. **10**, 775–782
38. Demeler, B., and Gorbet, G. E. (2016) Analytical ultracentrifugation data analysis with ultrascan-III. *Analytical Ultracentrifugation: Instrumentation, Software, and Applications*. 10.1007/978-4-431-55985-6_8
39. Brookes, E., Cao, W., and Demeler, B. (2010) A two-dimensional spectrum analysis for sedimentation velocity experiments of mixtures with heterogeneity in molecular weight and shape. *European Biophysics Journal*. **39**, 405–414
40. Brookes, E. H., and Demeler, B. (2007) Parsimonious regularization using genetic algorithms applied to the analysis of analytical ultracentrifugation experiments. *Proceedings of GECCO 2007: Genetic and Evolutionary Computation Conference*. 10.1145/1276958.1277035
41. Demeler, B., and Van Holde, K. E. (2004) Sedimentation velocity analysis of highly heterogeneous systems. *Anal Biochem*. **335**, 279–288
42. Min, C. K., Son, Y. J., Kim, C. K., Park, S. J., and Lee, J. W. (2011) Increased expression, folding and enzyme reaction rate of recombinant human insulin by selecting appropriate leader peptide. *J Biotechnol*. **151**, 350–356
43. He, W., Fu, L., Li, G., Andrew Jones, J., Linhardt, R. J., and Koffas, M. (2015) Production of chondroitin in metabolically engineered E. coli. *Metab Eng*. **27**, 92–100
